# Prolonged SARS-CoV-2 nucleic acid conversion time in military personnel outbreaks with presence of specific IgG antibodies

**DOI:** 10.1099/jmm.0.001498

**Published:** 2022-01-31

**Authors:** Jhonnatan Reales Gonzalez, Diego Prada Cardozo, Sheryll Corchuelo, Gabriela Zabaleta, Zonia Alarcón, Maria T. Herrera Sepulveda, Katherine Laiton Donato, Carlos Franco Muñoz, Diego A. Alvarez Diaz, Yesith Guillermo Toloza Perez, Ronald López, Jeadran Malagón Rojas, Giovanna Bresciani, Marcela Mercado

**Affiliations:** ^1^​ Grupo de Genómica De Microorganismos Emergentes. Dirección de Investigación en Salud Pública, Instituto Nacional de Salud, Bogotá, Colombia; ^2^​ Especialización en Estadística Aplicada, Fundación Universitaria Los Libertadores, Bogotá, Colombia; ^3^​ Grupo de Microbiología. Dirección de Investigación en Salud Pública, Instituto Nacional de Salud, Bogotá, Colombia; ^4^​ Grupo de Morfología Celular. Dirección de Investigación en Salud Pública, Instituto Nacional de Salud, Bogotá, Colombia; ^5^​ Grupo de Salud Ambiental y Laboral. Subdirección de Investigación en Salud Pública, Instituto Nacional de Salud, Bogotá, Colombia; ^6^​ Dirección de Sanidad, Armada Nacional de Colombia, Bogotá, Colombia

**Keywords:** Prolonged COVID-19, rectal swabs, nucleic acid conversion time, false-recovered, re-exposure, RNA shedding

## Abstract

Coronavirus disease 2019 (COVID-19) is transmitted person-to-person mainly by close contact or droplets from respiratory tract. However, the actual time of viral shedding is still uncertain as well as the different routes of transmission. We aimed to characterize RNA shedding from nasopharyngeal and rectal samples in prolonged cases of mild COVID-19 in young male soldiers. Seventy patients from three different military locations were monitored after recommending to follow more strict isolation measures to prevent the spread of the virus. Then, nasopharyngeal, rectal, and blood samples were taken. SARS-CoV-2 RNA was detected by RT-PCR and specific antibodies by chemiluminescent immunoassays. The median nucleic acid conversion time (NACT) was 60 days (IQR: 7–85 days). Rectal swabs were taken in 60 % of patients. Seven patients (10 %) were positive in nasopharyngeal and rectal swabs, and five (7.14 %) remained positive in rectal swabs, but negative in nasopharyngeal samples. Four patients (5.71 %) that had been discharged, were positive again after 15 days. No significant difference was found in nucleic acid conversion time between age groups nor clinical classification. Maintaining distancing among different positive patients is essential as a possible re-exposure to the virus could cause a longer nucleic acid conversion time in SARS-COV-2 infections.

## Introduction

Coronavirus disease 2019 (COVID-19) is caused by the *Betacoronavirus* SARS-CoV-2, a virus identified for the first time in the city of Wuhan, Hubei province, China [1]. This is a potentially fatal respiratory disease that has spread rapidly around the world, being a great challenge for public health on a global scale [2]. The disease is transmitted mainly during the initial stage of infection through respiratory droplets and direct contact with infected individuals [3]. Although the viral load of SARS-CoV-2 decreases after the onset of the disease in the respiratory tract, the period of RNA shedding or nucleic acid conversion time (NACT) may differ among infected patients [4, 5]. The duration of this viral NACT is usually 20 days or less, but this spreading phase can vary among patients and last much longer [6, 7]. Prolonged viral shedding is an important aspect since it has been associated with an increased risk of death [8]; however, factors that can cause prolonged NACT are unclear [4]. Understanding the parameters that influence this duration is crucial for developing and implementing control strategies and optimizing antiviral medication treatment in COVID-19 patients [9, 10]. Nucleic acid conversion time (NACT) is defined as the period from the onset of symptoms to the date of first-negative RT-PCR result of at least two consecutive negative test results [11, 12], and it is extremely important since cases that have not achieved the nucleic acid conversion can disperse the viral infection after its apparent recovery [8], so longer observation periods should be considered for certain groups of patients with COVID‐19 [11].

Alternative routes of transmission of SARS-CoV-2 raise concern as it would be another factor that can increase viral spread in the population. Some studies have characterized the presence of viral infectious particles in faeces [13], suggesting the elimination of this pathogen through the gastrointestinal system. There have been reports of COVID-19 patients with negative conversion in nasopharyngeal samples but still, continue testing positive by RT-PCR through another type of samples [12].

Studying the dynamics of viral elimination for SARS-CoV-2 in different contexts and settings is essential to allow better health responses, tracking and treating of infected cases to reduce patients’ death and control cases during the pandemic [9, 14]. Here, we performed a retrospective study involving prolonged NACT in nasopharyngeal and rectal samples from young soldiers with mild COVID-19 using clinical records, RT-PCR and serological results to determine the nucleic acid conversion time.

## Methods

### Ethical considerations

This study was reviewed and approved by the Institutional Ethical Committee at Instituto Nacional de Salud, Colombia (CEMIN-09–2020). All patients signed the informed consent form. Patients’ information was anonymized to protect their identity.

### Study population

This is a retrospective study, with consecutive non-probabilistic sampling. Patients from three different military locations that had a previously confirmed diagnosis for SARS-CoV-2 were included. They were divided into two observation groups according to their isolation conditions. The first group of patients (*n*=33) were soldiers placed in isolation at a hospital setting in Bogotá, Colombia after an outbreak in their military base. Patients were gradually quarantined when they tested positive; every 7 or 14 days they were tested by the hospital. As all patients were positive, they had been placed in reduced spaces (nine patients per room). The second group enrolled (*n*=37) consisted of soldiers who were in military camps in two different departments, Antioquia (ANT) and Valle del Cauca (VAC), Colombia. After a visit to those locations and due to the persistent positive results in both groups, we recommended that patients wear disposable surgical masks (daily replaced), improve cleaning and disinfecting from patients’ facilities and increase physical distancing between patients. One week later and after they followed the above-mentioned recommendations, nasopharyngeal swabs and blood samples were taken from every patient by our laboratory. Patients with two consecutive negative results by RT-PCR were informed and discharged, and those who remained positive were maintained under observation. After ten more days, new nasopharyngeal swabs and blood samples were collected, as well as rectal swab samples (Fig. S1, available in the online version of this article). Patients that remained positive were under observation until their discharge.

### Swab samples and molecular detection

Nasopharyngeal and rectal samples were collected using sterile nylon microbiological transport swabs (Improve, China) and preserved in 500 µl of viral transport medium (VTM) (Bovine serum albumin, salt mix, gentamicin, amphotericin) (Viral ad-bio, Annar Health Technology, Col) and stored to −70 °C. Viral RNA was extracted using a commercial kit (QIAamp Viral RNA Mini Kit, QUIAGEN, USA) following the manufacturer´s instructions. RNA samples were tested to detect the presence of SARS-CoV-2 through real time RT-PCR using the commercial kit 2019-nCoV:Real-Time Fluorescent RT-PCR (BGI Genomics, Beijing, China). Human RNase P was used as an internal control for the real time RT-PCR. Ct values under 38 for the two targets (ORF1ab and Gene N) were taken as positive, values higher than 40 as negative, and values between 38 and 40 were repeated for confirmation.

### SARS-CoV-2 immunoassays

Blood samples (7 ml) were collected by venipuncture sampling using siliconized vacuum tubes without additives and containing serum gel separator (Becton Dickinson and Company, USA) to evaluate presence of specific SARS-CoV-2 antibodies. Specimens were immediately centrifuged at room temperature at 1500 ***g*** for 10 min and serum were aliquoted in 2 ml cryovials and stored at −70 °C. Serum samples were analysed by chemiluminescent immunoassays (CLIA) using ADVIA Centaur XP system (Siemens, Germany) along with the ADVIA Centaur SARS-CoV-2 IgG (sCOVG) assay previously validated in our laboratory [15], which is intended for qualitative and semi-quantitative detection of IgG antibodies to SARS-CoV-2. Manufacturer’s instructions were followed. Results are reported as arbitrary index values or U/ml and as reactive (Index <1.00) or non-reactive (Index ≥1.00). Index values were used to calculate binding antibody units per ml (BAU ml^−1^) as recommended by the Who International Standard for anti-immunoglobulins [16] and reported by the manufacturer as follows: BAU ml^−1^=Index value×21.8.

### Statistical analysis

A bivariate analysis was performed using Chi square (*X^2^*) test to evaluate the association of epidemiological characteristics. A Cox proportional hazards model was performed with a type two simple censor on the right (being the event of interest getting a negative result by RT-PCR); associations and comparison between groups were done with Hazard Ratio (HR) with confidence intervals (IC) of 95 %. A Mann-Whitney U or Kruskal-Wallis test were carried out to compare *Ct* values and BAU ml^−1^ between groups. Analyses were carried out using R software (version 4.0.2).

## Results

### Epidemiological characteristics of patients

A total of 70 patients previously diagnosed with COVID-19 were included in this study. All were male soldiers with a median age of 21 years (interquartile range [IQR] 20–26; range 18–41), coming from three different locations (Table 1). Sixteen patients (22.85 %) were symptomatic at the beginning of the infection. Cough (7.14 %), odynophagia (7.14 %), and fatigue (4.28 %) were the most common clinical signs among those patients. No patients were transferred to the intensive care unit (ICU) nor needed major medical attention. All patients recovered and were discharged.

**Table 1.** Epidemiological characteristics of soldiers with prolonged SARS-CoV-2

Variable	All patients (*N*=70)	Group 1 Patients (*n*=33)	Group 2 Patients (*n*=37)
**Age (years), median (IQR)**	21 (20–26)	20.4 (18–27)	26.1 (18–41)
***Origin, n (%)***			
Bogotá	33 (47.14 %)	33 (100 %)	–
Antioquia	22 (31.42 %)	–	22 (59,45)
Valle del Cauca	15 (21.42 %)	–	15 (40,54)
***Clinical Classification, n (%)***			
Symptomatic	16 (22.85 %)	6 (18.18 %)	10 (27.02 %)
Asymptomatic	54 (77.14 %)	27 (81.81 %)	27 (72.97 %)
***Signs and symptoms, n (%)***			
Cough	5 (7.14 %)	1 (3.03 %)	4 (10,81 %)
Odynophagia	5 (7.14 %)	1 (3.03 %)	4 (10,81 %)
Fatigue	3 (4.28 %)	1 (3.03 %)	2 (5.40 %)
Fever	1 (1.43 %)	1 (3.03 %)	–
Dysgeusia	1 (1.43 %)	1 (3.03 %)	–
Anosmia	1 (1.43 %)	1 (3.03 %)	–
nd	3 (4.28 %)	–	3 (8.10 %)
***Comorbidities, n (%)***			
Smoker	4 (5.71 %)	3 (9.09 %)	1 (2.70 %)
Asthma	1 (1.42 %)	–	–
Diabetes	1 (1.42 %)	–	–
nd	3 (4.28 %)	–	3 (8.10 %)
***IgG antibody detection***			
Number of reactive cases (%)	67 (95.1 %)	33 (100 %)	34 (91.9 %)
BAU ml^−1^, median (IQR)	120.12 (58.97–192.88)	124.5 (22.89–201.89)	112.3 (10.9–173.53)
**Nucleic acid conversion time (days), median (IQR)**	60 (7–85)	50 (7–73)	63 (31–85)

IQR, Interquartile range; nd, No data.

In asymptomatic patients it was counted since the first positive result.

### Prolonged SARS-CoV-2 RNA shedding detection

**Fig. 1.**
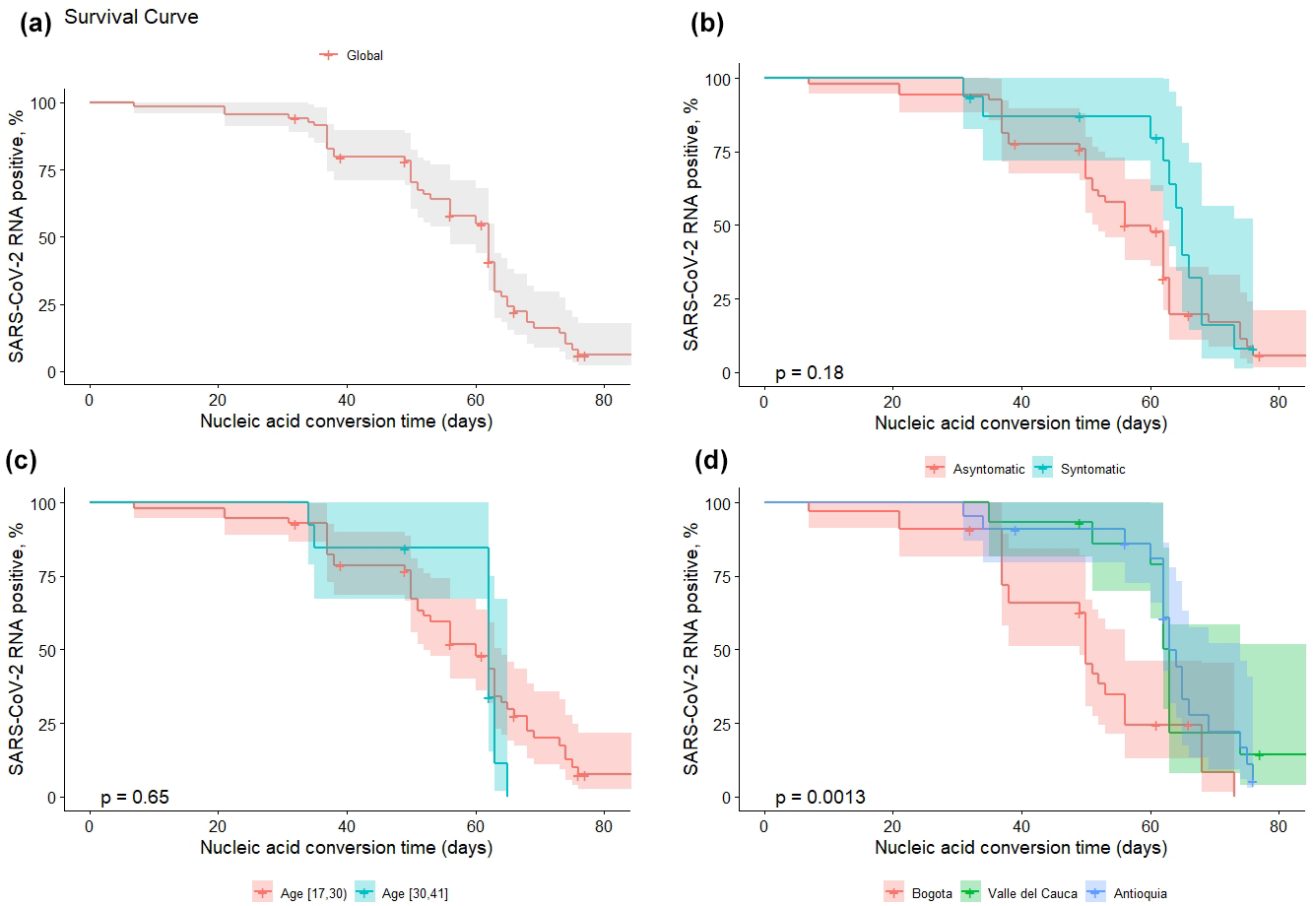
Kaplan-Meier curve of duration for patients with prolonged SARS-CoV-2 RNA shredding, *n*=70; 13 patients’ data were censored (**a**). Cumulative proportion of patients with detectable RNA since illness onset between symptomatic and asymptomatic patients (**b**), age groups (**c**), and patients’ place of origin (**d**).

All patients included were characterized for being RT-PCR positive for SARS-CoV-2 during a prolonged time. Although none of them had an acute disease and symptoms relieved few days after onset, RT-PCR tests from nasopharyngeal swabs remained positive. By the time it was informed to our laboratory, patients had a median of 49 days (IQR, 37–52 days) testing SARS-CoV-2 RT-PCR positive. After patients followed the given recommendations aiming to improve their isolation conditions and favour distancing among each patient (three patients per room), most of them turned SARS-CoV-2 RT-PCR negative between 7 and 10 days later, with a median of nucleic acid conversion time of 60 days (IQR, 43–63 days) (Fig. 1a). In the asymptomatic cases, NACT was calculated from first RT-PCR positive result. At the end of our follow-up 13 patients remained positive. However, until our last observation, these patients had a median of 61 days (IQR, 49–64 days) testing positive for SARS-CoV-2. These 13 patients were taken as censored in the survival analyses.

**Fig. 2.**
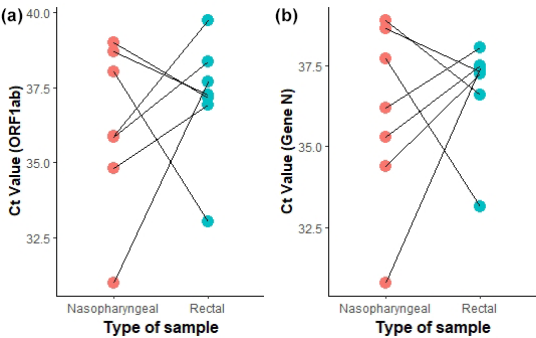
Comparison of *Ct* values for ORF1ab (**a**) and Gene N (**b**) in nasopharyngeal and rectal swabs samples. *Ct* values were compared for seven patients that tested positive in both samples at our last sampling. No significant difference in either target was found (*P*>0.05).

Taking into account the inadequate use of respiratory protection and reduced physical distancing between the patients that could result in contamination of the nasopharyngeal swab, as well as the reports of positivity in non-respiratory samples in patients with prolonged infection, rectal swabs were taken in a total of 42 patients (60%) to observe a potential prolonged faecal shedding of SARS-CoV-2 RNA. It was found that seven patients (10%) were positive by RT-PCR carried out from both nasopharyngeal and rectal swabs. When comparing *Ct* values from both type of swab samples, we found high *Ct* values for the two molecular targets (mean *Ct*=36.5) and no significant difference (*P=0.609*) (Fig. 2). Five patients (7.14%) showed negative results in nasopharyngeal swabs but remained testing positive in rectal samples and four patients (5.71%) that remained positive through nasopharyngeal swabs, showed negative results in rectal swabs (Table 2). Four patients that were discharged with our last sampling for being negative in both nasopharyngeal and rectal samples, tested positive again after 15 days, when another nasopharyngeal sample was taken, increasing their total RNA shedding time to 77 days. We also compared the *Ct* values from patients who remained positive in the last two samplings by our laboratory. Although there was a slight tendency to get higher *Ct* values throughout the time for the ORF1ab target (*P*=0.0273) (Fig. S2a), no significant differences were observed for Gene N *Ct* values (*P*=0.734) (Fig. S2b).

**Table 2.** Comparison between results of RT-PCR from nasopharyngeal and rectal samples (*n*=42)

	NP samples positive	NP samples negative	Total
**Rectal samples positive**	7	5	12
**Rectal samples negative**	4	5	9
**Total**	11	10	21

NP, Nasopharyngeal.

No significant difference was found in prolonged conversion time between age groups (*P=0.65*) nor clinical classification (symptomatic and asymptomatic) (*P=0.18*) according to Kaplan-Meier curves (Fig. 1b. c). Prolonged RNA shedding was statistically different between places of origin compared to Bogotá; VAC (HZ: 0.33; IC: 0.16–0.68; *P*=0.0027) and Antioquia (HZ: 0.314; IC: 0.17–0.59; *P*=0.0004) (Fig. 1d).

### IgG antibody levels against SARS-CoV-2 in prolonged cases

**Fig. 3.**
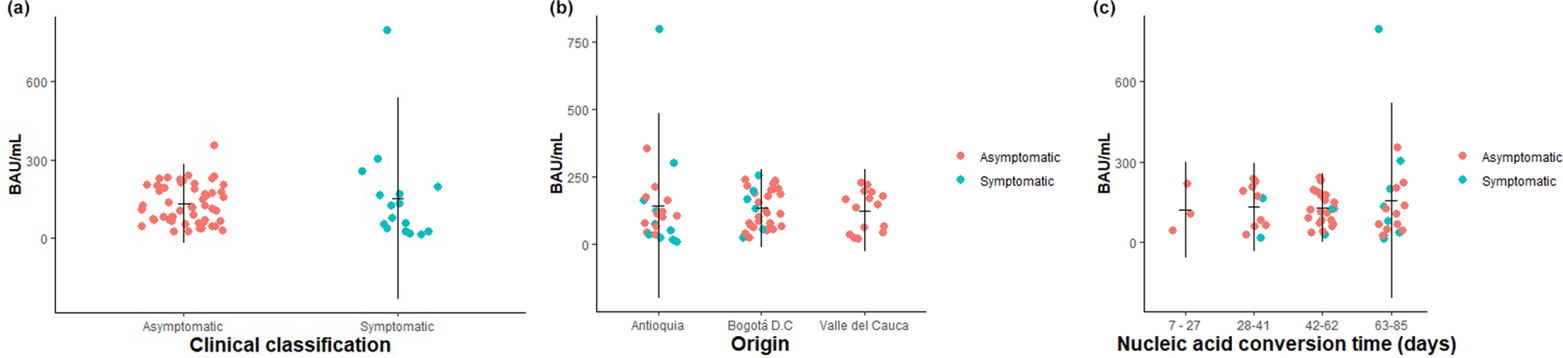
Comparison of IgG levels (BAU ml^−1^) between groups according to (**a**) clinical classification, (**b**) place of origin, and (**c**) nucleic acid conversion time. No significant differences were observed (*P*>*0.05*). Horizontal and vertical lines represent the mean value and standard deviation, respectively.

Presence of IgG antibodies against SARS-CoV-2 was detected in 67 patients (95.7%). The median value for the binding antibody units was 120.12 BAU ml^−1^ (Table 1). The time of blood collection since the onset of symptoms or first detection by PCR ranged from 0 to 60 days. IgG SARS-CoV-2 antibodies levels were compared between groups and no significant difference was found neither between symptomatic and asymptomatic patients (*P*=0.367) nor between places of origin (*P*=0.513). Similarly, no statistical differences were observed in patients when antibody levels were stratified according to the number of days for nucleic acid conversion (*P*=0.967) (Fig. 3).

## Discussion

Understanding viral shedding kinetics, contagiousness, and specific antibody reaction are essential to implement infection control strategies, assess the risk of transmission and guide decisions regarding isolation of patients [17, 18]. Although it is known that in most patients the SARS-CoV-2 nucleic acid conversion is around 21 days after illness onset [19], our findings are consistent with other reports of patients with a longer duration [18, 20], where SARS-CoV-2 RNA has been detected up to 105 days [5]. These results might suggest that SARS-CoV-2 has a relatively long replication period in some infected patients [21]. Lin *et al.*, identified longer nucleic acid conversion in COVID-19 patients as a risk factor associated to delayed discharged, so this measure is an important indicator for disease prognosis [12]. However, this data should be addressed carefully as false-negative results could show shorter nucleic acid conversion time. Almost all patients included in this study had developed antibodies for SARS-CoV-2, showing that viral RNA shedding continues even after seroconversion and absence of symptoms, supporting the found by Liu *et al.*, in one patient [17]. Three patients had not developed IgG antibodies, two were from ANT (symptomatic) and one from VAC (asymptomatic). This might be because the blood samples of these patients were taken during the first days after symptoms onset or first positive result by RT-PCR.

It has been reported that poorly ventilated and populated spaces favour viral transmission despite other preventive measures being taken [22], as a large number of aerosol droplets generated by simple activities as coughing or speaking, or fomite transmission, can result in a continuous infectious dose over the time [22, 23]. Studies report that reduced isolation spaces between confirmed patients with COVID-19 as military rehabilitation centres may bring a daily re-exposure and prolonged viral positivity [24], turning a problem for militaries worldwide [25]. During the first weeks after testing positive, these patients were isolated in reduced and poorly ventilated spaces without allowing an appropriate distancing among each other (nine patients per room). After they were separated in different rooms (three per room), and better cleaning of settings was adopted, most of the patients turned negative gradually. This might indicate that settings and conditions of isolation in positive SARS-CoV-2 cases are essential to end the infection or the viral RNA shedding, as these asymptomatic carriers could be a potential source of infection [24], so a definitive clearance can be harder to achieve if patients are in continuous exposure. This is consistent with our results and may be useful to determine isolation period and settings in asymptomatic and mildly symptomatic patients, especially in military institutions. This can also explain why the patients from VAC and Antioquia had a significant longer nucleic acid conversion time, as those groups of patients were isolated in camps in military locations and the Bogotá ones were in hospital facilities, therefore with better conditions. These military camps are settings with semi-enclosed environment with contained spaces in which many soldiers interact. These settings are known to facilitate a person-to-person transmission of disease agents and in turn are a problem for militaries worldwide [25]. Considerable efforts are required to control outbreaks, so measures to prevent and reduce transmission have to be implemented in populations at risk [26].

Factors associated with prolonged nucleic acid shedding have been assessed in patients with COVID-19. Results suggest that male sex is associated in such prolonged cases [27, 28]. More studies are needed as our sample studied just included men. Xiao *et al.*, also reported that RNA shedding tended to be more prolonged in older patients and in those that have comorbidities due to impaired immune function [21]. Here, we didn‘t find differences in the nucleic acid conversion time for two age groups; this might be because the group of older soldiers wasn’t old enough (30–41 years), and most of them didn’t have comorbidities.

Human-to-human transmission occurs mainly through respiratory droplets, but other routes have been investigated as SARS-CoV-2 can be detected in different types of samples [29]. The persistently positive RT-PCR on rectal swabs and faecal samples has been documented previously, suggesting a prolonged faecal viral excretion [30, 31]. Zhang *et al.*, found that during the initial stage of the infection most of the positive results were counted from oral swabs, but this appears to change in a later stage were more anal swabs tested positive [32, 33]. These results might support the idea of a transmission through fecal-oral route and indicate that viral shedding from the digestive system is greater and lasts longer than that from the respiratory tract [33]. SARS-CoV-2 has also been isolated and successfully cultured from stool samples of two patients without symptoms [34], so the prolonged positive detection of viral RNA from rectal samples might be the result of secretion of infectious virions from the infected gastrointestinal cells. Nevertheless, it is important to highlight the fact that a RNA detection does not mean the presence of viable virus nor an active infection in patients [35, 36]. Our results showed high *Ct* values for both type of samples in such prolonged cases. Bullard *et al.*, evaluate the relationship between RT-PCR *Ct* values and infectivity of SARS-CoV-2 in cell culture [37], finding that samples with *Ct* values higher than 24 were not able to infect *in vitro* and infectivity of patients with Ct >24 is low [37] or that RT-PCR is more sensitive to detect positive samples when compared to culture as proposed for other viral infections [38]. More studies for SARS-CoV-2 regarding this aspect should be considered. Likewise, testing different types of samples may improve the sensitivity and reduce false-negative test results [34].

We found that four patients who were discharged for testing negative by both types of samples, had positive results again after 15 days. This condition, more than a recurrence, can mean that a proportion of recovered patients may be virus carriers even if they are asymptomatic and show negative RT-PCR results, and that a possible intermittent viral shedding might occur in such patients [39, 40]. Therefore, it may be necessary a prolonged observation and repeat confirmation of detection tests for safe discharge of patients to avoid a wider spread of the infection [21]. On the other hand, this may be due to false negatives results of RT-PCR and/or prolonged nucleic acid conversion [11].

Among the limitations of this study, we highlight the fact that this was a retrospective study so we weren’t able to assess if the prolonged shedding was due to RNA or to viable virus spreading, which is necessary to confirm the potential fecal-oral transmission. However, based on additional studies previously done, we suggest that different type of samples, as rectal swabs, might be useful in determining the discharge of patients, especially in outbreak cases. Another limitation is that because of the nature of the study, we were not able to assess statistically if conditions at which patients were maintained before our recommendations to improve isolation, influence directly to prolonged RNA shedding. Nevertheless, more studies that evaluate these aspects are needed in order to help the decision makers regarding the conditions for isolating patients. SARS-COV-2 whole genome sequencing were performed to determine viral lineages causing the prolonged infections; however, it was no possible to obtain a minimum coverage value for lineage determination probably due to the low viral load in the samples (data not shown).

In conclusion, our results highlight the importance of maintaining distancing even among different positive patients, since a possible re-exposure to the virus could cause a longer nucleic acid conversion time in SARS-COV-2 infections even if patients have developed specific antibodies. This is essential in military institutions and persons deprived of liberty where quarantine or isolation settings often favour person-to-person transmission. Different routes of transmission should be taken into account in these populations as a discharge criterion based only on respiratory samples could lead to false-recovered patients.

## Supplementary Data

Supplementary material 1
