# Supplementary material for: Prolonged SARS-CoV-2 nucleic acid conversion time in military personnel outbreaks with presence of specific IgG antibodies
Source: J Med Microbiol. 2022 Jan 31;71(1):001498. doi: 10.1099/jmm.0.001498 (PMC8895548; doi:10.1099/jmm.0.001498)
Supplement: Supplementary material 1 [file jmm-71-1498-s001.pdf]

Table S1. Supplementary data. Additional information from included cases with epidemiological characteristics and antibody levels.

Patients	Origin	Gender	Age	Clinical classification	Symptons and signs	Clínical History/ Comorbidities	Time from symptons onset to first positive PCR (days)	Time from first Positive PCR to INS notification (days)	Nucleic acid conversion time (days)	Time from onset/first positive PCR to final follow-up*	Time from onset to Positive Results after first discharged (days)	Immunoassay Results	Immunoassay Interpretation	BAU/mL
Patient 1	Bogotá D.C	M	24	Symptomatic	Fever	None	ND	20	ND	32	NA	7,69	Reactive	167,6
Patient 2	Bogotá D.C	M	21	Asymptomatic	NA	None	NA	37	49	NA	NA	5,71	Reactive	124,5
Patient 3	Bogotá D.C	M	19	Asymptomatic	NA	Asthma	NA	37	ND	49	NA	2,37	Reactive	51,7
Patient 4	Bogotá D.C	M	21	Symptomatic	Anosmia,Dysgeusia, Odinophagia	None	ND	37	ND	49	NA	11,65	Reactive	254,0
Patient 5	Bogotá D.C	M	19	Asymptomatic	NA	Smoker	NA	54	ND	66	NA	9,26	Reactive	201,9
Patient 6	Bogotá D.C	M	22	Symptomatic	Cough, Fatigue/adynamia	None	0	49	ND	61	NA	2,57	Reactive	56,0
Patient 7	Bogotá D.C	M	19	Asymptomatic	NA	None	NA	49	ND	61	NA	9,42	Reactive	205,4
Patient 8	Bogotá D.C	M	19	Asymptomatic	NA	None	NA	49	ND	61	NA	3,18	Reactive	69,3
Patient 9	Bogotá D.C	M	18	Symptomatic	ND	None	9	52	73	NA	NA	1,05	Reactive	22,9
Patient 10	Bogotá D.C	M	27	Asymptomatic	NA	Smoker	NA	20	21	NA	NA	4,66	Reactive	101,6
Patient 11	Bogotá D.C	M	21	Asymptomatic	NA	None	NA	50	51	NA	NA	3	Reactive	65,4
Patient 12	Bogotá D.C	M	20	Asymptomatic	NA	None	NA	37	38	NA	NA	2,65	Reactive	57,8
Patient 13	Bogotá D.C	M	21	Asymptomatic	NA	None	NA	37	38	NA	NA	1,18	Reactive	25,7
Patient 14	Bogotá D.C	M	21	Asymptomatic	NA	None	NA	6	7	NA	NA	9,93	Reactive	216,5
Patient 15	Bogotá D.C	M	20	Asymptomatic	NA	None	NA	36	37	NA	NA	10,36	Reactive	225,8
Patient 16	Bogotá D.C	M	20	Asymptomatic	NA	None	NA	36	37	NA	NA	9,37	Reactive	204,3
Patient 17	Bogotá D.C	M	20	Asymptomatic	NA	None	NA	36	37	NA	NA	3,6	Reactive	78,5
Patient 18	Bogotá D.C	M	20	Asymptomatic	NA	None	NA	36	37	NA	NA	8,72	Reactive	190,1
Patient 19	Bogotá D.C	M	19	Asymptomatic	NA	None	NA	49	50	NA	NA	10,56	Reactive	230,2
Patient 20	Bogotá D.C	M	19	Asymptomatic	NA	None	NA	49	50	NA	NA	5,19	Reactive	113,1
Patient 21	Bogotá D.C	M	19	Asymptomatic	NA	None	NA	49	50	NA	NA	7,15	Reactive	155,9
Patient 22	Bogotá D.C	M	21	Asymptomatic	NA	None	NA	36	37	NA	NA	10,84	Reactive	236,3
Patient 23	Bogotá D.C	M	21	Asymptomatic	NA	None	NA	49	50	NA	NA	5,41	Reactive	117,9
Patient 24	Bogotá D.C	M	21	Asymptomatic	NA	None	NA	36	37	NA	NA	2,87	Reactive	62,6
Patient 25	Bogotá D.C	M	21	Asymptomatic	NA	None	NA	20	21	NA	NA	1,89	Reactive	41,2
Patient 26	Bogotá D.C	M	22	Asymptomatic	NA	None	NA	52	53	NA	NA	10,99	Reactive	239,6
Patient 27	Bogotá D.C	M	19	Asymptomatic	NA	None	NA	49	50	NA	NA	3,68	Reactive	80,2
Patient 28	Bogotá D.C	M	20	Asymptomatic	NA	None	NA	49	56	NA	NA	4,02	Reactive	87,6
Patient 29	Bogotá D.C	M	20	Asymptomatic	NA	None	NA	49	56	NA	NA	2,54	Reactive	55,4
Patient 30	Bogotá D.C	M	20	Asymptomatic	NA	Smoker	NA	45	52	NA	NA	8,17	Reactive	178,1
Patient 31	Bogotá D.C	M	20	Symptomatic	ND	None	12	49	68	NA	NA	8,99	Reactive	196,0
Patient 32	Bogotá D.C	M	18	Asymptomatic	NA	None	NA	49	56	NA	NA	8,52	Reactive	185,7
Patient 33	Bogotá D.C	M	21	Symptomatic	ND	Diabetes	15	46	68	NA	NA	6,05	Reactive	131,9
Patient 34	Antioquia	M	28	Asymptomatic	NA	None	8	64	76	NA	NA	1,99	Reactive	43,4
Patient 35	Antioquia	M	24	Symptomatic	ND	ND	26	36	66	NA	NA	3,42	Reactive	74,6
Patient 36	Antioquia	M	20	Asymptomatic	NA	None	0	35	ND	39	NA	7,39	Reactive	161,1
Patient 37	Antioquia	M	28	Symptomatic	ND	None	0	27	31	NA	NA	0,72	Non Reactive	NA
Patient 38	Antioquia	M	34	Symptomatic	Cough,Odinophagia	None	3	27	34	NA	NA	7,42	Reactive	161,8
Patient 39	Antioquia	M	21	Symptomatic	Fatigue/adynamia	None	4	56	64	NA	NA	13,87	Reactive	302,4
Patient 40	Antioquia	M	31	Asymptomatic	NA	None	1	57	62	NA	NA	4,88	Reactive	106,4
Patient 41	Antioquia	M	31	Symptomatic	Cough,Odinophagia	ND	2	57	63	NA	NA	36,58	Reactive	797,4
Patient 42	Antioquia	M	32	Symptomatic	ND	ND	1	57	62	NA	NA	5,67	Reactive	123,6
Patient 43	Antioquia	M	38	Symptomatic	Odinophagia	None	4	57	65	NA	NA	0,5	Non Reactive	NA
Patient 44	Antioquia	M	22	Asymptomatic	NA	Smoker	12	59	75	NA	NA	4,68	Reactive	102,0
Patient 45	Antioquia	M	24	Symptomatic	Cough,Fatigue/adynamia	None	13	59	ND	76	NA	2,41	Reactive	52,5
Patient 46	Antioquia	M	29	Asymptomatic	NA	None	0	70	74	NA	NA	3,04	Reactive	66,3
Patient 47	Antioquia	M	33	Asymptomatic	NA	None	16	42	62	NA	NA	7,96	Reactive	173,5
Patient 48	Antioquia	M	31	Asymptomatic	NA	None	16	42	62	NA	NA	3,6	Reactive	78,5
Patient 49	Antioquia	M	41	Asymptomatic	NA	None	16	42	ND	62	NA	5,15	Reactive	112,3
Patient 50	Antioquia	M	30	Asymptomatic	NA	None	2	57	63	NA	NA	5,61	Reactive	122,3
Patient 51	Antioquia	M	26	Symptomatic	Odinophagia	None	48	13	65	NA	NA	1,59	Reactive	34,7
Patient 52	Antioquia	M	19	Asymptomatic	NA	None	0	52	56	NA	NA	1,58	Reactive	34,4
Patient 53	Antioquia	M	20	Symptomatic	Cough	None	4	52	60	NA	NA	1,1	Reactive	24,0
Patient 54	Antioquia	M	19	Asymptomatic	NA	None	0	52	ND	56	NA	9,74	Reactive	212,3
Patient 55	Antioquia	M	26	Asymptomatic	NA	None	0	36	69	NA	NA	16,26	Reactive	354,5
Patient 56	Valle del Cauca	M	35	Asymptomatic	NA	None	0	45	ND	49	NA	1,09	Reactive	23,8
Patient 57	Valle del Cauca	M	19	Asymptomatic	NA	None	16	65	85	NA	NA	6,2	Reactive	135,2
Patient 58	Valle del Cauca	M	19	Asymptomatic	NA	None	5	65	74	NA	NA	2,95	Reactive	64,3
Patient 59	Valle del Cauca	M	20	Asymptomatic	NA	None	8	51	63	NA	NA	9,14	Reactive	199,3
Patient 60	Valle del Cauca	M	20	Asymptomatic	NA	None	8	51	63	NA	78	1,97	Reactive	42,9
Patient 61	Valle del Cauca	M	18	Asymptomatic	NA	None	8	51	63	NA	78	10,11	Reactive	220,4
Patient 62	Valle del Cauca	M	23	Asymptomatic	NA	None	8	51	63	NA	78	0,97	Non Reactive	NA
Patient 63	Valle del Cauca	M	19	Asymptomatic	NA	None	2	56	ND	77	NA	8,12	Reactive	177,0
Patient 64	Valle del Cauca	M	20	Asymptomatic	NA	None	7	51	62	NA	77	7,88	Reactive	171,8
Patient 65	Valle del Cauca	M	21	Asymptomatic	NA	None	7	51	62	NA	NA	8,89	Reactive	193,8
Patient 66	Valle del Cauca	M	19	Asymptomatic	NA	None	6	41	51	NA	NA	1,66	Reactive	36,2
Patient 67	Valle del Cauca	M	41	Asymptomatic	NA	None	16	42	62	NA	NA	10,42	Reactive	227,2
Patient 68	Valle del Cauca	M	30	Asymptomatic	NA	None	16	42	62	NA	NA	3,07	Reactive	66,9
Patient 69	Valle del Cauca	M	22	Asymptomatic	NA	None	14	42	60	NA	NA	6,79	Reactive	148,0
Patient 70	Valle del Cauca	M	33	Asymptomatic	NA	None	6	29	35	NA	NA	7,72	Reactive	168,296

Abbreviations

ND= no data

NA= Not applicable

*= Applies only for patients that didn´t turned negative until our final follow-up.

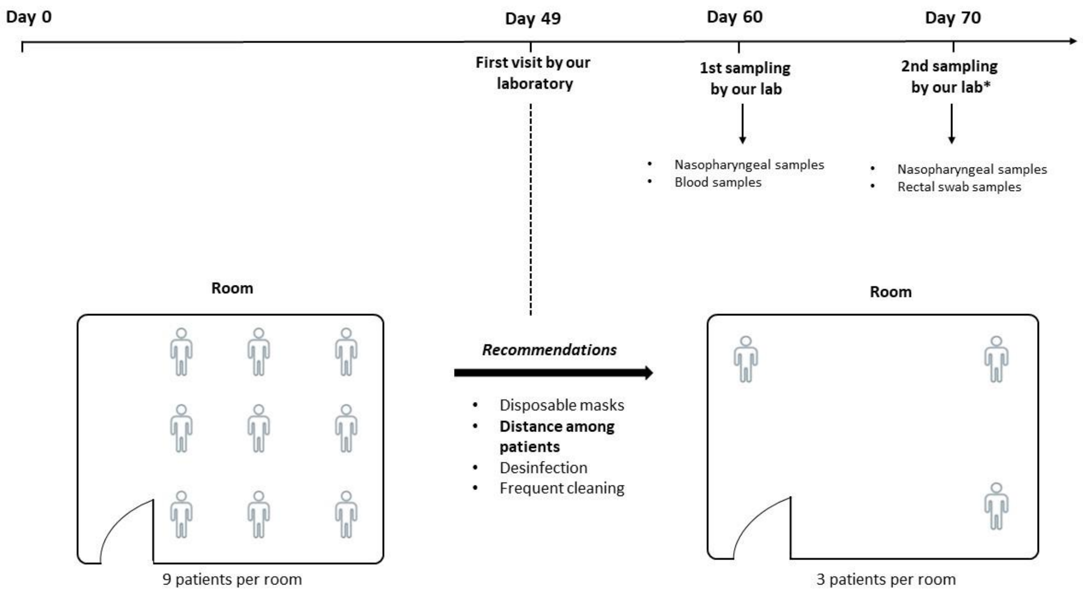

Figure S1. **Study sampling.** Patients were in average 49 days positive by RT-PCR when informed to our laboratory. We visit the setting conditions and recommendations were given to avoid cross-contamination among patients. Two samplings with ten days apart were taken after that.

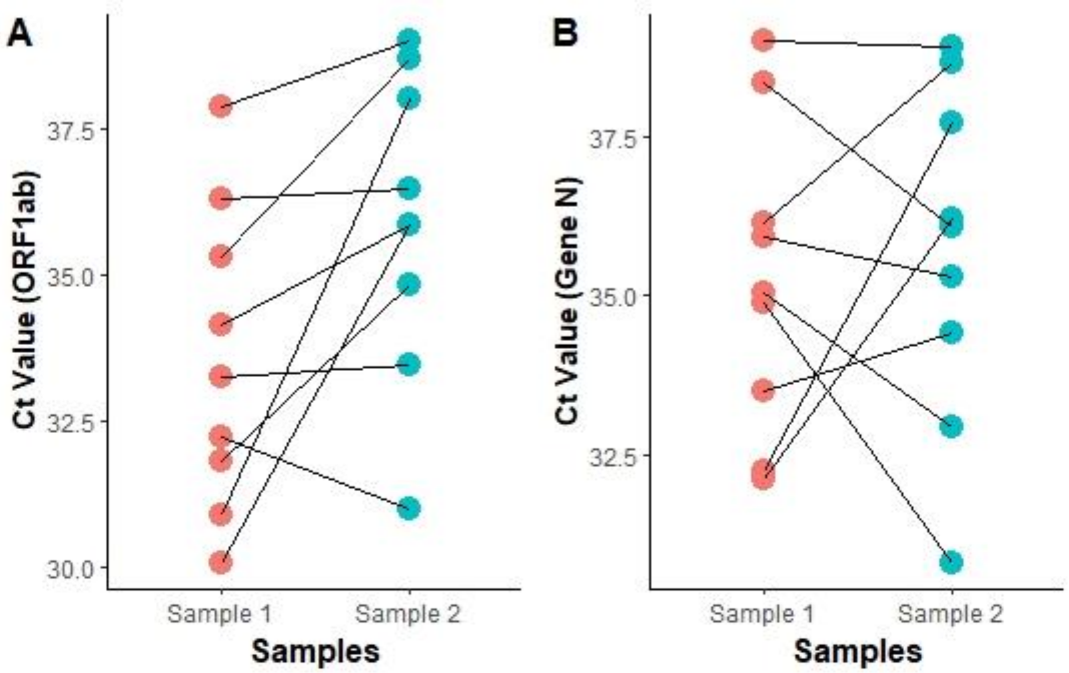

Figure S2. Comparison of *Ct* values obtained for ORF1ab (A) and Gene N (B) from samples taken ten days apart in remaining positive patients. Both respiratory samples were taken by our laboratory after recommendations were followed by patients. Statistical difference was observed for ORF1ab target (*p*=0.027), while it was not for Gene N (*p*=0.734).
